# An eHealth Platform to Manage Chronic Disease in Primary Care: An Innovative Approach

**DOI:** 10.2196/ijmr.4217

**Published:** 2016-02-09

**Authors:** Esther PWA Talboom-Kamp, Noortje A Verdijk, Lara M Harmans, Mattijs E Numans, Niels H Chavannes

**Affiliations:** ^1^ Public Health and Primary Care Department Leiden University Medical Centre (LUMC) Leiden Netherlands; ^2^ Saltro Diagnostic Centre Utrecht Netherlands

**Keywords:** eHealth, self-management, anticoagulation clinic, chronic obstructive pulmonary disease, venous thromboembolism, integrated disease management, chronically ill, telemonitoring, primary care

## Abstract

The number of individuals with chronic illness and multimorbidity is growing due to the rapid ageing of the population and the greater longevity of individuals. This causes an increasing workload in care, which results in a growing need for structural changes of the health care system. In recent years this led to a strong focus on promoting “self-management” in chronically ill patients. Research showed that patients who understand more about their disease, health, and lifestyle have better experiences and health outcomes, and often use less health care resources; the effect is even more when these patients are empowered to and responsible for managing their health and disease. In addition to the skills of patients, health care professionals need to shift to a role of teacher, partner, and professional supervisor of their patients. One way of supervising patients is by the use of electronic health (eHealth), which helps patients manage and control their disease. The application of eHealth solutions can provide chronically ill patients high-quality care, to the satisfaction of both patients and health care professionals, alongside a reduction in health care consumption and costs.

## Introduction

The average age of the Dutch population is increasing rapidly in two distinct ways. The entire so called “baby boomer” generation, born between 1945 and 1965, will have reached the age of 65 and enter the post-active phase of their lives within the next twenty years. Following this, the population size of future generations will be smaller. By 2025, 21% of the Dutch population will consist of citizens older than 65 years, compared to approximately 10% at the turn of the millennium [1]. In addition, the life expectancy of the Dutch population has increased in recent years. Between 1980 and 2012, the life expectancy for men increased by almost seven years (from 72.4 to 79.1 years), and for women by almost four years (from 79.1 to 82.8) [2]. Technological developments in medicine and health care, as well as improved treatment methods, are the keys to the earlier detection and more adequate treatment of chronic diseases. As a result, older people are living longer despite their chronic diseases. Due to a combination of these developments, close-to-home primary health care is increasingly dominated by relatively old patients with one or more chronic diseases. On account of the resulting capacity implications for primary care, organizational health care processes will now have to be reviewed. Furthermore, new technologies will have to be tested and introduced, and it will be necessary to establish whether patients' care needs can be better managed by promoting their own sense of responsibility.

We subscribe to the new definition of health by Huber et al [3]; health is no longer defined as a static situation but as the ability to adapt and to self-manage, in the face of social, physical, and emotional challenges. In this definition, self-management is an important and irreplaceable part of health and disease management.

From this perspective, chronic diseases require lifestyle changes and an approach that is referred to as “self-management”: the ability to actively participate in the management of health with the emphasis on complete physical well-being. This involves medical management; changing, maintaining, and creating meaningful behaviors and dealing with the emotions of suffering from chronic disease(s) [4].The most important skills for self-management are problem solving, decision making, resource utilization, and taking action. The basic principle underlying self-management is that behavioral change cannot succeed without the patient taking responsibility [5].

In addition to skills of patients, another necessary ingredient for self-management is a good relationship between the patient and health care professional [4]. Until the first half of the 20th century, health care professionals were trained to diagnose and treat diseases. With the introduction of self-management, this role changed to being a teacher, partner, and professional supervisor. One way of supervising patients is by use of electronic health (eHealth), which helps patients manage and control their disease. The application of eHealth solutions can provide chronically ill patients high-quality care, to the satisfaction of both patients and care professionals, alongside a reduction in health care consumption and costs. One way of supporting self-management is the introduction of eHealth.

The pressure to implement self-management through eHealth is immense as the number of individuals with chronic illness and multimorbidity is growing fast, due to the rapid ageing and greater longevity of the population. The growing number of individuals suffering from major chronic illnesses faces many obstacles in coping with their condition, not the least of which is medical care that often does not meet their needs for effective clinical management, psychological support, and information [6]. Cumulatively, chronic diseases are the leading cause of death in many developed countries with cardiovascular and respiratory diseases dominating death statistics. Between 2005 and 2025, the number of heart failure and chronic obstructive pulmonary disease (COPD) cases in the Netherlands is expected to each rise by approximately 100,000, an increase of 45% and 33%, respectively [1].

## The Case of Chronic Obstructive Pulmonary Disease

The World Health Organization (WHO) estimates that over 210 million people currently suffer from COPD. Three million people died worldwide from the disease in 2005. Although a change in smoking habits may alter this slowly, by 2020, COPD is expected to be the third most common cause of death worldwide [7]. Due to the increasing prevalence and complex treatment involved, COPD will account for a significant increase in health care costs, as well as for a growing capacity problem in care. In 2007, the number of COPD patients in the Netherlands was 276,100; between 2005 and 2025, this number is expected to increase by 38% [8].

Patients with COPD account for a higher consumption of care resources than people without COPD. On average, they visit their general practitioners (GPs) 12.7 times per year, of which 2.1 times are for COPD. In contrast, other people visit their GPs 6.1 times per year [8]. In 2005, the total cost for COPD- and asthma-related patient care was estimated at €799 million, placing COPD and asthma in the top ten of the most expensive diseases [5,8].

The two early stages of COPD, The Global Initiative for Chronic Obstructive Lung Disease (GOLD) 1 and 2 [9], represent 80% of the total COPD population in the Netherlands. These patients are mainly examined and treated within primary care. In the years to come, more and more patients with COPD will be referred back to primary care from secondary and tertiary care. Primary care has ample intervention options to offer patients with COPD that may lead to improvement of their condition. These include reactivation by support of physical therapists, smoking cessation programs, and self-management supported by bronchodilator medication. Various programs containing elements of these interventions have been implemented and tested for effectiveness in primary care [10]. A number of initial positive effects have been published so far, showing that these programs result in clinically relevant improvement in the areas of dyspnoea, exercise tolerance, and quality of life after one year [10-12].

It is well known that smoking cessation and exercise programs, as part of a multidisciplinary approach, are the most effective treatments for COPD [13]. Integrated Disease Management (IDM) programs for patients with COPD promoting self-management and exercise result in improved disease-specific quality of life and exercise capacity, and a reduction in hospital admissions and days spent in hospital [10,14]. However, this multidisciplinary approach is difficult to organize in primary care, and has, therefore, mainly been implemented and tested for effectiveness in secondary and tertiary care. Due to the organizational approach within the current health care processes, such programs have not been implemented for longer periods of time and have not produced intrinsic motivation on the part of patients to permanently switch to a healthy and active lifestyle. The main challenge within the next few years will be to strengthen the patients' own role in a responsible manner. Research has shown that self-management leads to better treatment of COPD; patients are more likely to adjust their lifestyles once they have actually acquired a sense of involvement in their disease. Fear of hospitalization and passive behavior hinder the early detection of exacerbations [15]. Effing et al demonstrated that self-management education leads to a reduction in hospital admissions and fewer sick days resulting from exacerbations [16]. Bourbeau et al showed that the application of self-management programs by patients with severe COPD results in a 40% reduction in hospital admissions [17]. Individual action plans and proper disease education for patients with moderately severe COPD improved the level of recognition and self-treatment of severe exacerbations; hence, the impact on the patients' health status due to exacerbations was reduced while promoting recovery [18]. In the bigger picture, effective self-management programs for patients with COPD may contribute to better quality of life and to a reduction in health care consumption [19], as well as health care costs [20].

An important success factor in several COPD self-management trials was that the self-management program had been effectively integrated into a disease management program, with a continuing and more remotely positioned role for health care professionals [21-23].

A few studies have been performed on eHealth interventions for patients with COPD [24-27]. While these studies mainly focused on the economic effects, they provided evidence of a decrease in the number of visits to the hospital, resulting in cost reduction. Pinnock et al examined the effectiveness of telemonitoring COPD parameters integrated into existing care programs; this intervention had no impact on the rate at which patients with COPD were admitted to the hospital. The quality of the telemonitoring process may not have sufficiently enabled patients to actually take control and the authors themselves suggest that the existing care process insufficiently improved during the study [28].

## The Case of Venous Thromboembolic Disorders

Venous thromboembolism (VTE) is a common cause of potentially preventable mortality, morbidity, and high medical costs [29]. With ageing populations and persisting unhealthy lifestyles, the prevalence of VTE is rising rapidly [30]. Between 2005 and 2009, the number of patients with VTE in the Netherlands increased by 13%. In 2009, there were more than 385,000 patients with VTE in the Netherlands, more than half of whom suffered from atrial fibrillation [31]. Treatment of VTE consists of, among other interventions, anticoagulant therapy (AT) with vitamin K antagonists (VKA) to slow down the formation of blood clots [30]. AT requires frequent monitoring of the extent to which the blood clots, as well as regular visits to an anticoagulation clinic, laboratory, or physician, for venous puncture and analysis.

For this group of patients, it can be hypothesized that self-management (self-testing and self-measurement) might increase the sense of involvement in their own care. In recent years, various methods have been implemented and tested for measuring the degree of anticoagulation (international normalized ratio (INR)) in the home setting by means of self-measurement equipment. A meta-analysis by the Cochrane Collaboration in 2010 found that self-management (including self-dosing) by AT patients at home in combination with VKA treatment resulted in a decrease in thromboembolic complications and mortality at a constant frequency of bleeding complications [32]. This also applies to the Dutch situation with its extensive network of well-organized anticoagulation clinics [31].

Structured clinical trials with online self-management show a greater improvement in INR values within the therapeutic range (10%-23%) than self-management studies without online support (improved time in therapeutic range (TTR) less than 4%) [33,34]. Home measurement of INR and the reporting and dosing of weekly results online increase the TTR from 72% to 79% compared to conventional computer-assisted monitoring in an anticoagulation clinic [35]. Patient satisfaction proved to be higher using online remote monitoring of INR [36].

In anticoagulation clinics, it has been reported that fewer thromboembolic complications are reported if the self-management program is embedded in well-organized thrombosis care from a central thrombosis control center integrated in primary care [37,38].

## Self-Management and eHealth

The changing and growing demand for care is causing health care costs to spiral upward in the Netherlands [5]. At the same time, there is an imminent shortage of professional health care workers, estimated to be between 280,000 and 800,000 in the Netherlands in 2025 [39]. These two aspects combined are increasing the pressure on health care, while at the same time compromising quality, accessibility, and sustainability. To ensure the provision of proper health care, a rearrangement of duties is required. “Traditional care” is reactive, mainly focused on the treatment of episodes of disease or derailment. However, changing care demands call for a more proactive policy. This can be achieved by the timely detection of diseases or complications and by continuously structured monitoring of patients for care gaps and adverse changes in their condition to ensure a faster response to changes and complications. Another element of a proactive policy consists of giving patients themselves a prominent role in coping with their illness and well-being [22,40].

The rising number of chronically ill patients and increasing workload in care bring along a growing need for structural change within the health care system. Based on this perspective, in recent years the focus has mostly been on promoting self-management in chronically ill patients. In doing so, the objective is to give patients a more prominent role in dealing with their disease and sense of well-being; self-management is not only a convenient way to organize care differently, but also offers patients significant benefit. By providing patients with more knowledge about their disease and by active involvement in the process, patients are better able to accept and maintain a healthier lifestyle [41]. The effect is even more when these patients are empowered to and responsible for managing their health and disease [42].

Offering chronically ill patients innovative self-management solutions, such as eHealth, can support or even improve their independence. Many options exist for patients to get involved through websites and platforms; the quality and content vary greatly, as do the results [43].

Several studies have shown that based on this approach, patients are better able to cope with their illness at the time and place of their choosing, allowing them to better adapt their lifestyle to their condition while taking some of the burden off the medical staff [44]. The deployment of eHealth facilitates the accessibility to health care, which in turn enhances the patients' understanding of their disease, sense of control, and willingness to engage in self-management [45,46]. By applying eHealth solutions, chronically ill patients can be provided with high-quality care, to the satisfaction of both patients and health care professionals [47,48].

The results of eHealth-supported self-management depend on the patients' expectations and level of education. Beenkens, for instance, asked 485 patients in anticoagulation clinics why they had opted for eHealth [49], and it appeared that patients mainly expect to gain benefits in their well-being, for example in the form of less travel and waiting time, and more freedom of movement. This study also showed that highly educated patients are more inclined to adopt eHealth than those with a low level of education [49].

Research into self-management in patients with COPD showed that more relevant positive effects are measured in the group of “effective self-managers”, predominantly characterized by relatively younger age, cardiac comorbidity, relatively more serious complaints, and living with others [50,51].

The Whole System Demonstrator (WSD) program is a large, randomized trial in England, in which 238 GP practices offered 6191 chronically ill patients various forms of telehealth or standard care. The telehealth systems in this study were designed to monitor vital signs, symptoms, and self-management behavior. The telehealth services were integrated within the existing GP practices and compared with a control group that was offered standard care.

An evaluation after one year showed lower mortality rates and fewer acute admissions in the group using telehealth than observed in the standard care control group [52]. It is possible that these differences were partly caused by an initial temporary increase in acute admissions in the control group. In another WSD evaluation, no differences were found between the telehealth group and the standard care group, measured by quality of life, anxiety, and depression symptoms [53].

Based on the initial results from the WSD program it can be assumed that patients receiving telehealth services are less likely to go for treatment at an accident and emergency department; further research is required to determine the underlying mechanism. Furthermore, anxiety and depression did not increase among patients using telehealth.

The randomized controlled trial (RCT) by Pinnock et al yielded the conclusion that the integration of telemonitoring within existing care had no effect on delayed hospitalization, on health-related quality of life, anxiety and depression, self-efficacy, and knowledge [28]. In their analysis, they argue that the added value generated by the WSD program can be partly explained by a general improvement in the quality of care, as a side-effect of implementation of telehealth [28].

## The eVita COPD and PORTALS Studies

Based on the available knowledge described, we formulated two research questions that we wish to answer using data from our large-scale implementation projects (Textbox 1). In these projects we will record and evaluate the effects of eHealth interventions within integrated primary care in the two mentioned domains of chronic disease primary care-managed COPD (eVita COPD), and anticoagulant therapy in venous thromboembolic conditions (PORTALS).

**The two questions we aim to answer.**

- What is the effect of the kind of eHealth implementation on use of the portals and patient outcomes?
- Does the effect depend on (1) subjectively experienced practical added value for patients, thereby making their everyday lives easier? and (2) The level of organization as an integral part of existing care?

We designed the multi-level study e-Vita to investigate different implementation methods of a self-management Web portal to support and empower patients with COPD in three different primary care settings; the level of integration of the Web portal within the care program is different in the three settings. Using a parallel cohort design, the clinical effects of the implementation of the Web portal will be assessed using an interrupted times series (ITS) study design and measured according to changes in health status with the Clinical COPD Questionnaire (CCQ). The different implementations and net benefits of self-management through eHealth on clinical outcomes with be evaluated from human, organizational, and technical perspectives. To our knowledge, eVita is the first study to combine different study designs that enable the simultaneous investigation of clinical effects (changes in health status), as well as effects of different implementation methods whilst controlling for confounding effects of the organizational characteristics.

We also used a parallel cohort design for the anticoagulation clinic patients in the PORTALS study. In this study, patient self-testing and patient self-management (including a Web portal) will be offered to patients of a thrombosis service who currently receive usual care for long-term AT. To investigate determinants of optimal implementation, we will compare two different implementation methods (1) after inclusion where participants will be randomly divided in subgroups where one group will be trained and educated by e-learning, and (2) the other group that will receive face-to-face group training. A third group, the non-self-management group consists of patients who continue to receive regular care.

In this PORTALS study, we will compare clinical outcomes and self-management skills of two different implementation methods. Second, the relationship between self-management skills, clinical outcomes, and individual characteristics will be investigated.

## Hypotheses

On the basis of earlier eHealth research, we expect to see problems where patients' motivation is concerned when it comes to starting and continuing to use the patient platform [54]. If patients use the self-management platform on a regular basis, we expect to see a positive effect on quality of life, complications, and hospitalization rate in both groups.

For patients with COPD, we expect to see a relatively small improvement in their everyday lives using the digital platform. Resulting from this, we assume that the use of the platform will grow and take root less rapidly.

Patients with VTE are linked to a center that determines their INR values on a regular basis, following which the clinic determines the dose of their medication. This process has far-reaching effects on their daily lives. For these patients, a comprehensive self-management program supported by a digital platform will ease their dependence on the anticoagulation clinic and enhance their sense of self-reliance. Therefore we expect these patients to use the digital platform more frequently. As a result, we expect even better improvements in both clinical outcomes and quality of life for patients with VTE.

## The Potential Added Value of eHealth

It is difficult to draw general conclusions about the impact of eHealth. The evidence of clinical and structural effects of eHealth interventions in patients with COPD and VTE is not clear-cut, partly because of the large differences in study design, interventions, and research methods. Furthermore, research methods into eHealth are a regular topic of discussion, as the focus on clinical outcomes often masks other beneficial effects.

Chronic diseases require lifestyle changes and an approach that is referred to as self-management: the individual ability to properly deal with symptoms, treatment, and physical and social consequences. The basic principle underlying this approach is that behavioral change cannot succeed without the patient taking his or her responsibility [5]. eHealth is a useful method to implement self-management.

The rising number of chronically ill patients and increasing workload in care bring along a growing need for structural change within the health care system. Using eHealth as a method to implement self-management can provoke beneficial effects for both patients and caregivers.

We designed the studies eVita COPD and PORTALS, both parallel cohort designs with Web-based support for self-management, where we expect to see a positive effect on clinical outcomes and quality of life of patients through the implementation of a self-management patient platform integrated within primary care. We presume that behavioral change in both patients and caregivers is the basis for these positive effects. The implementation of eHealth will support caregivers to have a better coaching relationship with their patients and the use of eHealth will help patients take a more leading role towards their own health status and lifestyle.

## Abbreviations

AT: anticoagulant therapy
COPD: chronic obstructive pulmonary disease
eHealth: electronic health
GP: general practitioner
INR: international normalized ratio
ITS: interrupted times series
TTR: time in therapeutic range
VKA: vitamin K antagonists
VTE: venous thromboembolism
WSD: Whole System Demonstrator program
